# Energetics and Structural Characterization of the large-scale Functional Motion of Adenylate Kinase

**DOI:** 10.1038/srep08425

**Published:** 2015-02-12

**Authors:** Elena Formoso, Vittorio Limongelli, Michele Parrinello

**Affiliations:** 1 Department of Chemistry and Applied Biosciences, ETH Zurich, and Faculty of Informatics, Institute of Computational Science, Università della Svizzera Italiana, via G. Buffi 13, CH-6900 Lugano, Switzerland; 2 Kimika Fakultatea, Euskal Herriko Unibertsitatea (UPV/EHU) and Donostia International Physics Center (DIPC), PK 1072, 20080 Donostia, Euskadi, Spain; 3 Università della Svizzera Italiana (USI), Faculty of Informatics, Institute of Computational Science, via G. Buffi 13, CH-6900 Lugano, Switzerland; 4 Department of Pharmacy, University of Naples “Federico II”, via D. Montesano 49, I-80131 Naples, Italy

## Abstract

Adenylate Kinase (AK) is a signal transducing protein that regulates cellular energy homeostasis balancing between different conformations. An alteration of its activity can lead to severe pathologies such as heart failure, cancer and neurodegenerative diseases. A comprehensive elucidation of the large-scale conformational motions that rule the functional mechanism of this enzyme is of great value to guide rationally the development of new medications. Here using a metadynamics-based computational protocol we elucidate the thermodynamics and structural properties underlying the AK functional transitions. The free energy estimation of the conformational motions of the enzyme allows characterizing the sequence of events that regulate its action. We reveal the atomistic details of the most relevant enzyme states, identifying residues such as Arg119 and Lys13, which play a key role during the conformational transitions and represent druggable spots to design enzyme inhibitors. Our study offers tools that open new areas of investigation on large-scale motion in proteins.

Cellular homeostasis is preserved through finely regulated molecular mechanisms, some of them involving macromolecules called metabolic monitors. In particular, these systems control the cellular energy state by generating signaling molecules that counteract energy unbalancing through the stimulation of specific molecular targets.

One of these metabolic monitors is adenylate kinase (AK). This enzyme coordinates different signaling pathways, ensuring adequate response to a broad range of functional, environmental and stress stimuli. In such a way, AK plays a key role in the cell and its dysfunction is connected to the onset of several human diseases, such as heart failure, metabolic disorders, cancer and neurodegenerative diseases^1^,^2^.

From the biochemical point of view, AK enzyme catalyzes the reversible nucleotide phosphoryl exchange reaction MgATP+AMP↔MgADP+ADP, controlling the cellular energy supply by regulating the ratio between AMP and ADP/ATP^3^,^4^. This reaction is made feasible by the structural organization of AK and collective domain motion on the μs–ms timescale^5^. In fact, AK has three domains, called CORE, LID and NMP, and two distinct binding sites (Figure 1). Specifically, ATP, that is complexed with Mg^+2^ ion, is bound between the CORE and LID domains, in the so called ATP binding site, while AMP is sandwiched between the CORE and NMP domains, in the AMP binding site. The CORE domain is conformationally stable during the action of the enzyme, while LID and NMP domains perform large-scale conformational changes^6^. These motions have both a structural and functional role^7^,^8^. In fact, they form the catalytic site suitable for the phosphoryl transfer reaction and at the same time shield the binding sites from waters, thus preventing the ATP and AMP hydrolysis^9^. Once the two ADP molecules are formed, the enzyme opens the LID and NMP domains releasing the products. Despite all this information, an exhaustive elucidation of the molecular mechanism of the motion between the different AK states is missing. This description is of paramount importance since it offers the opportunity for an exogenous control of the enzyme activity.

In this regard, the structural resolution of the open and closed forms of AK^10^,^11^ has motivated numerous experimental and theoretical studies on the molecular mechanism of this enzyme^12^,^13^,^14^,^15^,^16^,^17^,^18^. In particular, kinetic experiments have suggested that the enzyme conformational changes are the rate-determining step of the catalytic reaction, thus confirming the functional role of these motions^5^,^19^,^20^,^21^. In line with these studies, experiments using single-molecule fluorescence resonance energy transfer^22^ and ^15^N-NMR^23^ have shown that after phosphoryl transfer from ATP to AMP, the conformational motion from the closed to the open conformation is the rate-determining step of the reaction that produces ADP. In addition to these experiments, many theoretical works have investigated the AK functional mechanism^7^,^15^,^24^,^25^,^26^,^27^. Despite the efforts and the use of atomistic and multi-scale models, these simulations were not able to provide a complete scenario of the enzyme functional mechanism due to different limitations. For instance, in some cases the simulation time was too short^25^,^27^, while in others the presence of Mg^+2^ was neglected^7^,^25^. These approximations led to large discrepancies in the energy difference estimate between the different conformational states^7^,^24^.

In this scenario, many details of the AK functional mechanism remain unclear and a complete picture of the whole process is still lacking. For instance, the order in which the ligands bind is not entirely clarified, although a random binding mechanism is commonly accepted^20^,^28^. Furthermore, there are contradictory models describing the conformational changes carried out by the enzyme. Some authors suggest an independent motion of the domains^29^,^30^, while others propose a concerted one^7^,^12^,^15^,^31^. In this context, information regarding the coordination of the domain motions or the existence of transition and intermediate states during the AK action, would represent an important breakthrough.

Using methods developed in our group^32^,^33^,^34^,^35^, we are in the position to perform a more exhaustive conformational sampling of the AK functional motions overcoming the limits of previous investigations. In particular, using well-tempered metadynamics simulations (WT-MetaD)^34^ combined with path collective variables (path CVs)^35^, we have performed simulations of the opening and closing motion of the LID and NMP domains in the AK apo form. Recently, WT-MetaD and path CVs have proven to be successful in sampling large protein scale motion in many different case studies^36^,^37^. In particular, we biased the path CV that describes the motion of the LID domain from the closed to the open state (see Methods). Using this protocol, in ~1 μs of enhanced sampling simulations we were able to reproduce the µs-ms timescale collective motion of AK and characterize at atomistic level the key aspects of the AK functional transitions. Furthermore, combining WT-MetaD with path CVs, we are able to reconstruct the free-energy landscape of the investigated process, identifying the lowest free-energy path that connects the different states visited by the system. In addition to thermodynamics, we provide the fully atomistic description of the system during the functional transitions. In particular, we found that the enzyme samples a wide ensemble of conformations differing in free energy only by a few k_B_T. We identified residues such as Arg88, Arg119 and Lys13 that are involved in the enzyme conformational changes as potential druggable spots. This detailed description of the events complements the picture coming from previous studies, revealing new structural information on the AK functional mechanism of great value to guide drug discovery strategies.

## Results

As discussed in the introduction, the available structural data show that in AK the LID and NMP domains undergo the major conformational changes^6^. Thus, it can be suggested that while the opening of the two domains favors the binding of the ligands, their closure locks the enzyme in the catalytic competent conformation. Once the phosphoryl transfer reaction is completed, the two domains open up again to release the products.

**Figure 2:**
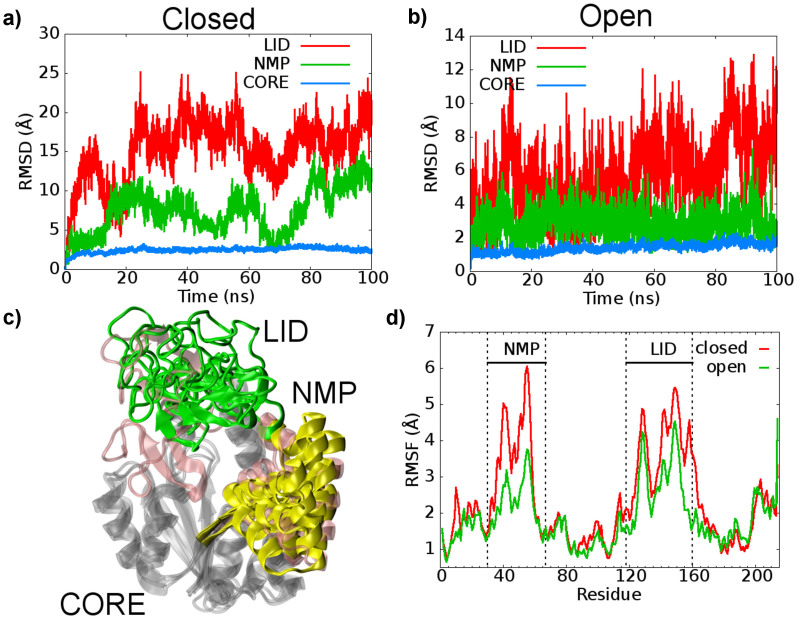
Molecular Dynamics (MD) simulations on AK. (a) and (b) rmsd values calculated for each domain of AK in the closed and open states undergone to standard MD simulations. All the structures were aligned using the CORE domain's Cα atoms. The LID is depicted in red, NMP is depicted in green and CORE is shown in blue. (c) Superimposition of selected conformations from the MD simulations with the X-ray closed and open states. The LID and NMP are colored in green and yellow cartoon, respectively, while the CORE in grey. The X-ray LID and NMP conformation are represented in pink. (d) Root mean square fluctuation (rmsf) of the backbone atoms calculated for the closed (in red) and open (in green) AK conformation during standard molecular dynamics simulations.

We decided to validate this hypothesis performing 100 ns long standard MD simulations for the closed and open states of AK in the ligand-free state. As shown in the rmsd and rmsf plots (see Figure 2), the major conformational changes occur at the LID and the NMP domains as expected. It is interesting to note that in both the simulations AK visits conformations far from the closed X-ray conformation (see Supporting Information Figure S1). This finding is suggestive that the binding of the ligands is necessary to stabilize the fully closed conformation of the enzyme. Furthermore, we found that starting from the closed state, the opening of the LID and NMP domains is observed within a relatively short time (see Supporting Information Figure S2). This event suggests that such motions occur in a short time scale, in line with previously published results^7^,^12^,^22^,^48^. However, the difficulties in describing all the AK conformational changes, that take longer time, prompted many groups to use coarse-grained models^15^,^28^. Unfortunately, such multi-scale approaches can miss important atomistic information, such as the formation and the dissociation of specific residue interactions. Using metadynamics we are in the position to overcome these limitations and the temporal barriers that have limited previous studies while keeping full atomistic resolution.

We discuss in detail the results obtained from the WT-MetaD simulations in the following paragraphs.

### Functional mechanism

Using metadynamics in ~1 μs of sampling we were able to reproduce the μs-ms timescale collective motion of AK. During the metadynamics simulations the major conformational changes occur at the LID and the NMP domains, as expected (see Supporting Information Figures S3 and S4). More detailed information on the specific conformations assumed by the LID and the NMP domains can be obtained from the FES computed as a function of CVs different from that originally biased. This is possible using the reweighting algorithm of Bonomi et al.^33^ (see Methods for details).

**Figure 3:**
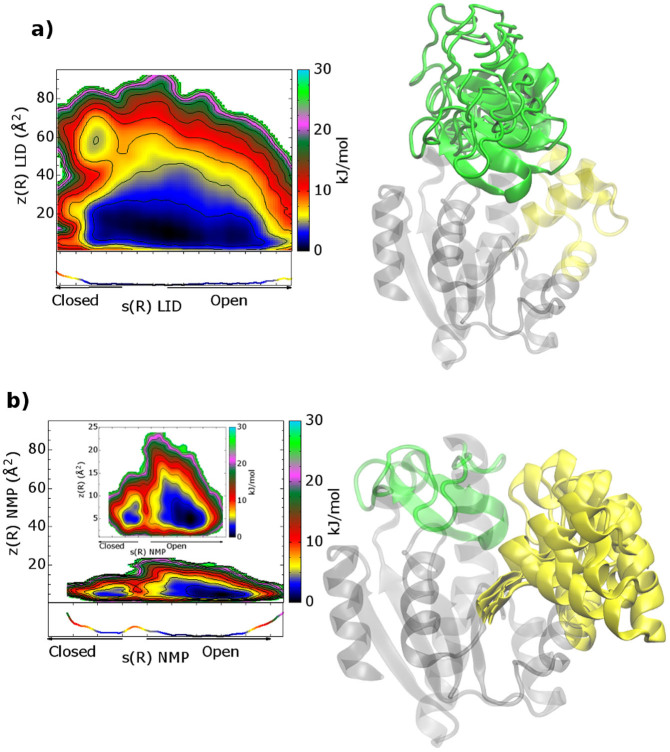
FESs associated with the conformational transition pathway of AK. Isosurfaces are shown each 2.5 kJ/mol. a) The FES as a function of the LID path CVs s(R) and z(R) (see Methods for details). b) The FES as a function of the NMP path CVs s(R) and z(R) (see Methods for details). To give an impression of the motion we superimposed different frames from the lowest free-energy paths for LID (a) and NMP (b). The LID and NMP domains are depicted in green and yellow, respectively.

**Figure 4:**
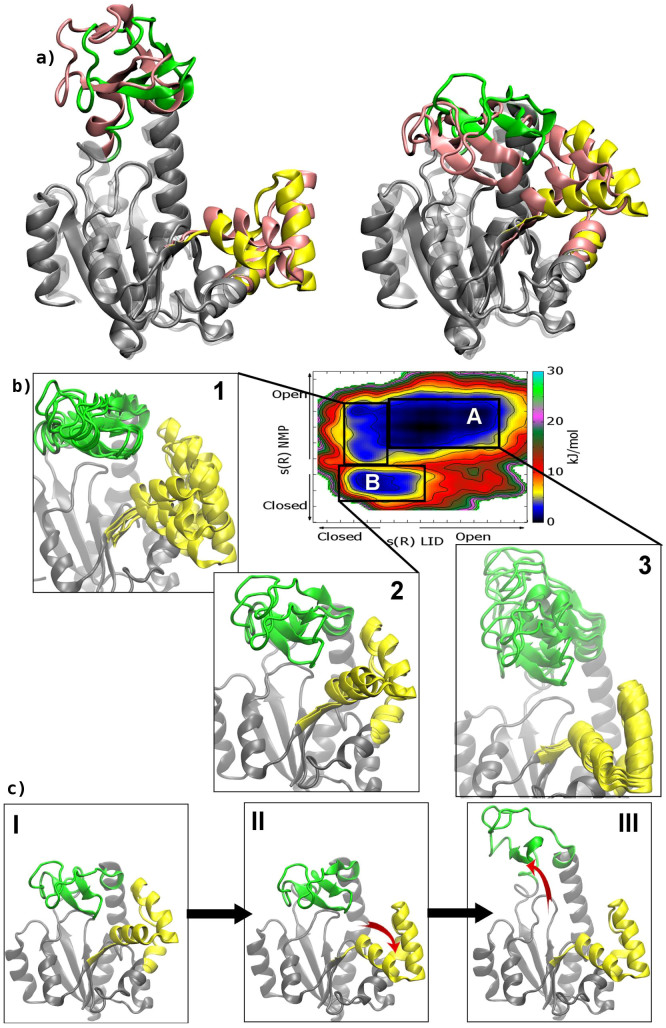
Characterization of the AK functional mechanism. (a) The molecular structures corresponding to the main free-energy minima A (open conformation) and B (closed conformation) states. The conformations are superimposed on the open and closed X-ray structures^11^,^21^. The LID and NMP domains are colored in green and yellow, respectively for the free-energy minima states and in pink for the X-ray structures. (b) The FES of the AK closed/open transition is shown as a function of LID domain closed to open transition (x axis) and NMP domain closed to open transition (y axis). Isosurfaces are shown each 2.5 kJ/mol. Different conformations of LID and NMP domains are represented in insets 1 to 3. The LID and NMP domains are depicted in green and yellow, respectively. (c) The lowest free-energy path of AK opening is represented in insets I to III. The LID and NMP domains are depicted in green and yellow, respectively.

In particular, in the FES computed as a function of the rmsd values relative to the AK open and closed X-ray structures (see Supporting Information Figure S5), it is clear that the enzyme visits several conformations, which are intermediates between the open and closed states and are separated by only a few k_B_T. Furthermore, comparing the FESs computed as a function of the path CVs s and z for each domain (see Figure 3 and Methods for details), one can note that the conformational space explored by the LID domain is wider than that visited by NMP. It is also relevant to stress that the relatively low z values explored in both FESs indicate a good choice of the reference path. In our simulations, the LID domain is more flexible than the NMP domain, assuming several conformations ranging from the crystallographic open^21^ to the closed^11^ structure, and exploring also conformations far from the original path (Figure 3). All these states are equally possible having similar energy values. In particular, one can note that the LID domain motion presents a broad single-well profile, represented by different isoenergetic conformations. At variance with LID, the NMP domain motion is very close to the reference path, and the closed and open states are separated by a free-energy barrier of ~ 4 k_B_T (Figure 3). However, the energetically most stable state of the enzyme presents the LID and NMP domains in an open conformation (Figure 4). In particular, the open state (A basin) is 1-2 k_B_T lower in free energy than the closed one (B basin), in line also with previous umbrella sampling calculations^24^ (Figure 4b). The higher energy stability of the open state is to be expected, since this conformation is functional to the binding of the ligands. Furthermore, it is interesting to note that the B conformation is similar but not identical to the fully ligated closed X-ray state (Figure 4a). In fact, in this pose the LID domain is slightly rotated towards the NMP domain, where α-helix 2 assumes a semi-closed conformation with respect to the X-ray structure (Figure 4a and S6). This difference is due to the fact that the X-ray closed form of AK was obtained in presence of an inhibitor mimicking the two physiological substrates (PDB ID code: 1AKE). This ligand influences the conformation of the LID domain since the latter interacts directly with the inhibitor. As our simulations are performed on the ligand free form, the LID domain cannot interact with the ligand and it is found closer to the NMP domain.

Our results are also instrumental to provide significant insights into the sequence of AK motion. In particular, looking at the FES shown in Figure 4b, one may note that the lowest free-energy path that connects the NMP open state to the closed one, shows the LID domain in a semi-closed conformation. On the other hand, when the NMP domain is closed, the LID domain is able to visit conformations that range from closed to semi-open states (inset 2 in Figure 4b). These findings suggest that the NMP domain opens first, followed by the LID (Figure 4c). In fact, the full opening of the LID domain is possible only when the NMP domain is in the open conformation. Furthermore, while the LID motion is barrier free, the NMP domain has a relatively high free-energy barrier between the open and closed states, ~ 4 k_B_T (Figure 3a and 3b). These findings indicate that the rate-delimiting step for the functional conformational changes of AK is the motion of the NMP domain.

A movie showing the AK motion under the action of metadynamics is reported in the Supporting Information.

To verify the stability of the A and B free-energy minima conformations we carried out over 100 ns unbiased MD simulations for each state. As regards pose A, the protein is more flexible with the LID domain fluctuating among several conformations in the open state. It arrives till the semi-closed position, but most of the time is fluctuating in the open state. Instead, in pose B the protein is stable for 28 ns, then the LID domain moves to the semi-open conformation. This behavior is in line with the metadynamics results (Figure 4b) showing a wider FES region for the A minimum and with the previously reported MD results.

### Structural mechanism

**Figure 5:**
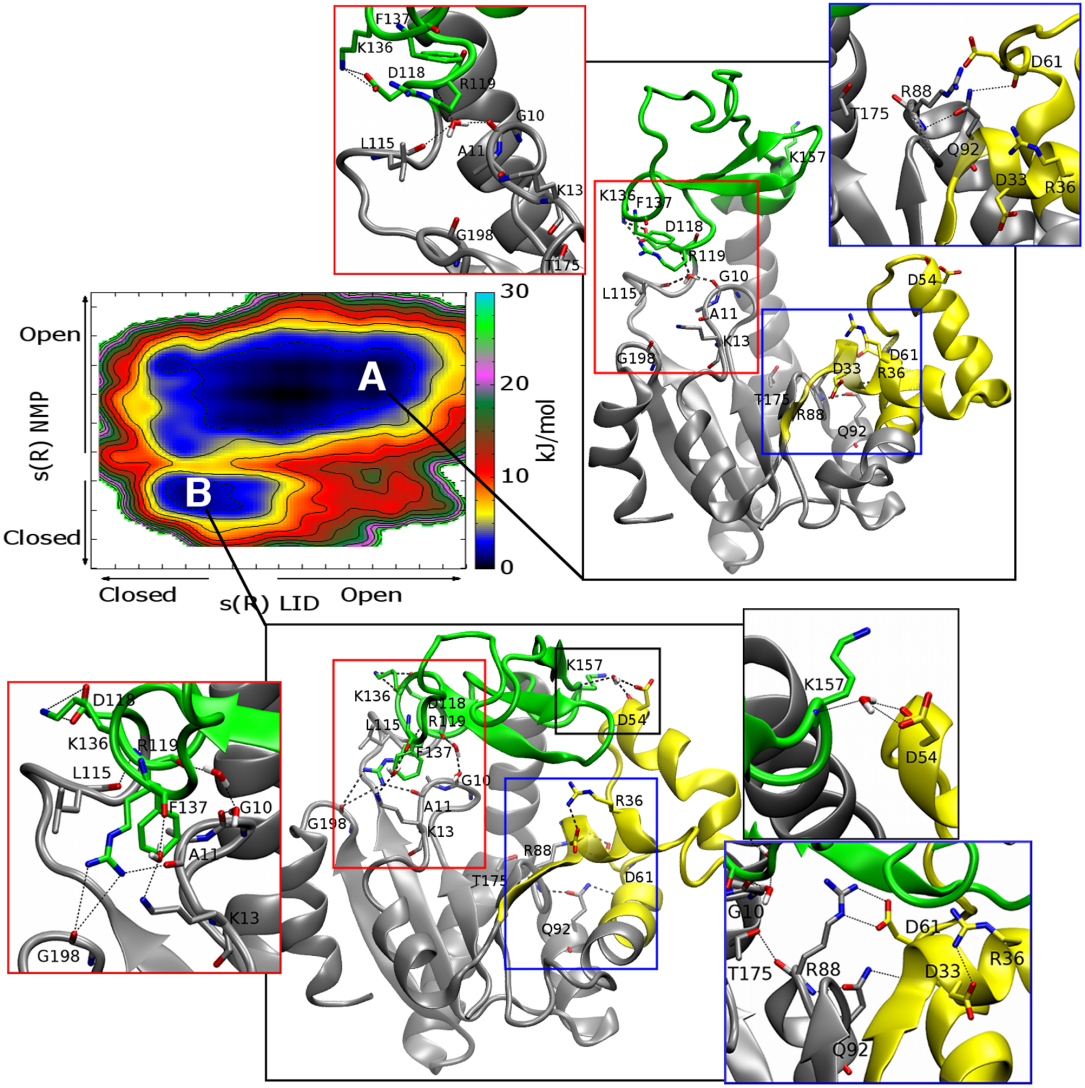
Atomistic description of AK functional motions. The FES of the AK closed/open transition is shown as a function of LID domain closed to open transition (x axis) and NMP domain closed to open transition (y axis). Isosurfaces are shown each 2.5 kJ/mol. The inter-residue interactions established at the main free-energy minima A and B are highlighted. The LID and NMP domains are colored in green and yellow, respectively. H-bonds are displayed as black dashed lines, while hydrogens are omitted for clarity reason.

From the metadynamics simulations we can also obtain atomistic information on the AK opening and closure mechanism. This analysis is necessary to elucidate the enzyme functional mechanism and provide the structural bases to develop AK drug-like ligands. We do this by first looking at the arginines that are close to the active site and that are conserved in the adenylate kinase family. To this we are also prompted by a number of experimental evidences suggesting a functional role of these residues during catalysis^11^,^39^. Most of these arginines engage strong interactions with the surrounding residues in the AK active site. During the transition from closed to open state, the side chains of three of these conserved arginines (Arg36, Arg88 and Arg119) become more flexible and do not form any favorable contact (Figure 5). In particular, Arg36, situated in the NMP domain at α-helix 2, loses the interaction with Asp33 after the closed to open transition (blue insets in Figure 5). Arg88, which is situated in the CORE domain and is involved in the binding of AMP to the catalytic site^40^, has in the closed conformation new partners such as the carboxylate group of Asp61 (α-helix 4) and residue Thr175 (α-helix 7), further stabilizing this state (blue insets in Figure 5). Finally, Arg119, in the closed conformation H-bonds with the carbonyl groups of Ala11 (P-loop) and Gly198, while at the same time it forms a cation-π interaction with Phe137, a residue of the LID domain (red insets in Figure 5). Moreover, the H-bond network formed by Gly10, Leu115 and Arg119, is mediated by a water molecule in the open conformation, while it is replaced by direct interactions in the closed state (red insets in Figure 5). It is worth mentioning that in the fully ligated form of the enzyme, Arg119 favors the orientation of ATP ligand in the binding site. Thus, given the pronounced flexibility displayed during the enzyme conformational change by Arg88 and Arg119, targeting these residues and their molecular partners could represent a suggestive strategy to block the enzyme in a specific conformation.

A behaviour similar to that of residues Arg88 and Arg119, was also observed for Lys13 situated in the nucleotide binding loop (GXXGXGK), which is known as P-loop and is highly conserved throughout the different forms of AK. In fact, in the open conformation Lys13 is very flexible and does not form favorable contacts (red insets in Figure 5). Instead, in the closed form this residue interacts via water molecules with the carbonyl group of Phe137 in the LID domain, stabilizing the closed state of this domain. It worth mentioning that Lys13 is conserved in the AK protein family and in the fully ligated form of the enzyme it contributes to orient the phosphate groups of the ligand in the catalytic site^41^. This information makes this residue a druggable spot to interfere with the AK functional mechanism. This consideration is further supported by the evidence that the Lys13/Gln mutated form of AK was found to be inactive^42^.

In addition to these interactions, both the energy minimum conformations A and B show the LID domain stabilized by H-bonds between Asp118 (α-helix 6) and Lys136 (LID domain) (red insets in Figure 5). The role of this interaction is rather debated in literature. In fact, some authors suggested that this interaction stabilizes only the open state of the LID domain^25^, while others have very recently shown the presence of this interaction also in the AK closed state^38^. However, we point out that for the apo form we find a closed configuration slightly different from the fully ligated one and our observation is a consequence of this fact. Other interactions that play a role in stabilizing the closed state are the water-bridged interactions between the backbone of Lys157, a residue of the LID domain, and the backbone or side chain of Asp54, a residue of the NMP domain at α-helix 3 (black inset in Figure 5).

The presence of many water-mediated interactions underlines the necessity of using explicit solvent simulations to describe accurately all the interatomic contacts ruling the AK functional transitions. Finally, although no H-bond between the opposite ends of the P-loop are present, the integrity of the loop is conserved in all simulations.

## Discussion

In biology many proteins exert their activity assuming different conformations and passing from the active to inactive state. Here, we have presented a thorough investigation with all-atom simulations of the conformational changes in Adenylate Kinase enzyme. In particular, we simulated the opening and closing motion of the Adenylate Kinase's LID and NMP domains, using well-tempered metadynamics simulations combined with path collective variables. Our protocol allowed estimating the free energy of the different protein states identifying the lowest energy path connecting the closed and the open states. In the open state we found that AK can assume a number of different conformations. The free energy is more favorable in this open conformation and the flexibility of the open state is functional to the capture of the ligand. In the closed state the structure assumes a conformation different from the X-ray one, which was crystallized in association with the ligand. Upon binding of ATP and AMP, the free-energy landscape of the AK conformational motion could sensitively change, responding to the different tasks performed by the enzyme. A series of computations are underway to shed light on these aspects of the AK functional mechanism in the presence of the ligands. Our simulations also characterize the mechanism of opening of AK showing that the LID domain can reach the open state only after the NMP one. This finding suggests that the motion of the NMP domain is the rate-determining step of the enzyme opening mechanism. Furthermore, the solvent has been found to play a fundamental role stabilizing the energy minimum conformations, therefore it must be taken into account explicitly.

This study provides a series of thermodynamics and structural information of great value to guide further computational and experimental investigations on this system. For instance, in drug design one might exploit the inter-residue interactions identified in our simulations to design compounds able to interfere with such interactions and block the enzyme into a specific state. If this succeeds the enzyme activity is inhibited and the designed ligands will have great potentialities as therapeutic agents for the treatment of heart failure, metabolic disorders, cancer and neurodegenerative diseases.

## Methods

### Molecular Dynamics

All the MD simulations of closed and open ligand-free AK systems were performed in explicit solvent using ff99sb amber force-field^43^,^44^, TIP3P water model^45^ and periodic boundary conditions in the GROMACS 4.5.3 MD package^46^. The crystal structures of open and closed AK were obtained from the protein data bank, PDB code 4AKE^21^ and 1AKE^11^, respectively. The X-ray structure of the closed conformation was resolved in complex with the inhibitor P^1^, P^5^-bis(adenosine-5′-)pentaphosphate (Ap_5_A), that works mimicking the two physiological substrates. Thus, the starting structure for the simulation of the closed apo form was obtained by removing that inhibitor from the X-ray structure.

The systems were solvated with ~17700 water molecules in a cubic box of 82.7 Å^3^ and neutrality was obtained by adding 4 Na^+^ ions. The standard Amber partial charges were applied to all the enzyme's and waters' atoms and ions. The long-range electrostatic interactions were computed by using particle mesh Ewald method (PME)^47^,^48^ in combination with a switch function for the direct-space part. A cutoff of 10 Å for the direct-space part, 72 FFT grid points for each of the lattice directions and fourth-order B-spline interpolation for spreading the atomic charges to the FFT grid were used. Nonbonded interactions were cut at 10 Å and shifted so as to smooth the Lennard-Jones term. All bonds were constrained using LINCS algorithm^49^, and the time step of the simulations was 2 fs. A steepest descent minimization was followed by 2 ns canonical ensemble equilibration and by another 2 ns isobaric-isothermal ensemble equilibration with Bussi thermostat^50^ and Berendsen barostat at 300 K and 1 atm.

### Well-tempered Metadynamics with Path Collective Variables

The PLUMED plugin (v1.2.2)^32^ was used to carry out metadynamics^51^ calculations with the GROMACS 4.5.3 code^46^. Using this technique a bias potential is added on a number of selected degrees of freedom, called collective variables (CVs). This operation enhances the sampling allowing to describe long time scale events, from microseconds to milliseconds, in a reasonable computational time (e.g. hundreds of nanoseconds). At the end of the simulation the free-energy surface of the process under investigation can be computed using the added bias. More in details, metadynamics (MetaD)^51^ consists of an adaptive scheme where Gaussian-shaped repulsive potentials are deposited along the simulation in the space of the chosen degrees of freedom (CVs). The history-dependent bias potential is made up by the sum of these deposited Gaussians and is added to the Hamiltonian of the system. In such a way, the system is discouraged to sample states already visited, thus accelerating the sampling. Here we used the well-tempered version^34^ of metadynamics^51^, which has proven to be successful in sampling long timescale motion in many biologically relevant systems^52^,^53^. Using this formalism the height of the added potential is decreased along the simulation to improve convergence of the FES. At the end of the MetaD simulation, the bias potential compensates the underlying free-energy surface (FES) and is related to the free energy following the formula: 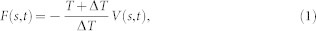
where *V(s,t)* is the bias potential added to the system, *F(s,t)* is the estimated FES as a function of the CVs at time *t* and *T* is the temperature of the simulation. Δ*T* is an input parameter with the dimension of a temperature and it is proportional to the energy barrier. Thanks to this formalism, one can increase barrier crossing and facilitate the exploration in the CVs space by tuning Δ*T*. In our simulations, Gaussian potentials of initial strength of 2 kJ/mol were deposited every 2 picoseconds and they were gradually decreased on the basis of the adaptive bias with a Δ*T* of 3,300 K.

In order to describe the opening and closing of the LID and NMP domains, we used the path CVs^35^. The path CVs are extremely powerful whenever one wants to study a transition between two states A and B. Given a reference path that connects A and B states, path CVs are flexible descriptors that represent the progression along the path and the distance from it. Specifically, let S(R) be a reduced representation of a generic configuration R. If the choice of S is appropriate, we would expect the reactive trajectories to be bundled in a narrow tube around the path. The path is described with a discrete number of frames S(l)^54^. To trace this path, we have followed the procedure of Branduardi et al.^35^ introducing the two variables s(R) and z(R): 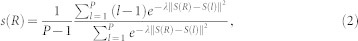

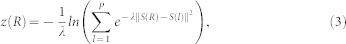
which measure the intercept and the distance of a microscopic configuration R from the reference path S(l), respectively. P is the number of frames that define S(l), ||S(R)-S(l)||^2^ is calculated as the mean square displacement after optimal alignment and λ is proportional to the inverse of the mean square displacement between successive frames.

### LID domain's path

In the present case, the reference path for the transition of the LID domain coincides with the geometrical interpolation from the closed to the open AK conformation. The path was obtained by a linear interpolation between the X-ray closed^11^ and open^21^ conformations of the enzyme using the morphing routine (g_morph) of the GROMACS package^46^. The rmsd of the Cα atoms of selected residues of the LID and CORE domains is calculated after alignment on selected LID and CORE atoms (see Supporting Information Table S1). We verified that the set of configurations obtained was equally spaced in the adopted mean square displacement metrics, and the value of λ was chosen to be comparable to the inverse of the mean square displacement between successive frames, 225 nm^−2^.

As starting conformation for the metadynamics simulations we used the enzyme equilibrated structure obtained from the previous classical MD simulations on the AK closed state. Metadynamics calculations were performed in the space of s(R), which is the variable that represents the conformational path connecting the open to the closed state of the LID domain, while z(R) was constrained to z(R) < 1 nm^2^. This gives the possibility for the system to explore conformations different from the original path, while maintaining at the same time the system reasonably close to the chosen intermediate frames. In such a way, the loss of the secondary structure is avoided. The hill width for s(R) was chosen to be 0.03. This value was chosen after measuring the fluctuations of s(R) in standard MD simulation.

### NMP domain's path

The reference path for the transition of the NMP domain coincides with the spatial interpolation from the closed to the open AK conformation. Also in this case, the path was obtained by a linear interpolation between the X-ray closed^11^ and open^21^ conformations of the enzyme using the morphing utility (g_morph) of the GROMACS package^46^. The rmsd of the Cα atoms of selected residues of the CORE and NMP domains is calculated after alignment on selected NMP domain's residues and all the CORE domain's Cα atoms (see Supporting Information Table S1). The value of λ was chosen to be comparable to the inverse of the mean square displacement between successive frames, 146 nm^−2^. The path connecting the NMP closed to the open conformation was defined by two variables s(R) and z(R) following the procedure of Branduardi et al.^35^ as previously described.

We note that no bias was added on these CVs, which were instead used to perform post-processing analysis of the simulation. Once the calculations converged, the FES was calculated along this path CVs using the reweighting algorithm developed by Bonomi et al.^33^.

The figures were rendered using the VMD software^55^ while the graphs were generated using gnuplot.

## Author Contributions

V.L. and M.P. designed the work, E.F. performed calculations, E.F., V.L. and M.P. analyzed the results and wrote the manuscript.

## Supplementary Material

Supplementary Information Supplementary Information

Supplementary Information Supplementary video

## Figures and Tables

**Figure 1:**
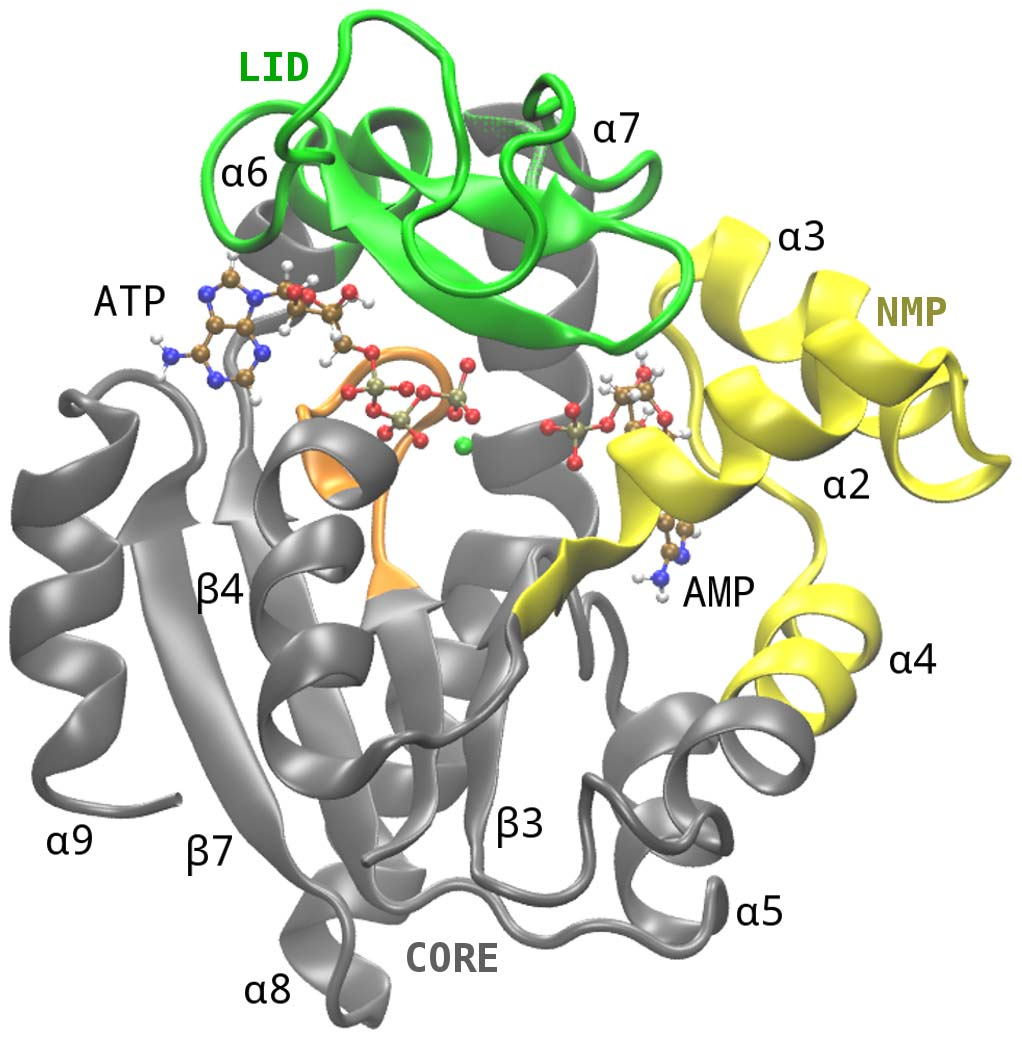
Structure of Adenylate Kinase. AK has three domains, called CORE, LID and NMP, and two distinct binding sites. The LID and NMP domains are colored green (residues 118–160) and yellow (residues 30–67), respectively. The CORE domain is colored in gray (residues 1–29, 68–117, 161–214) and the P-loop is colored in orange (residues 7–13). Specifically, ATP, that is complexed with Mg^+2^, is bound between the CORE and LID domains, in the so called ATP binding site, while AMP is sandwiched between CORE and NMP, in the AMP binding site. ATP and AMP ligands are represented as ball and stick.

## References

[b1] Dzeja P. P., Bast P., Pucar D., Wieringa B. & Terzic A. Defective Metabolic Signaling in Adenylate Kinase AK1 Gene Knock-out Hearts Compromises Post-ischemic Coronary Reflow. J. Biol. Chem. 282, 31366–31372 (2007). 17704060 10.1074/jbc.M705268200 PMC3232003

[b2] Corrons J. V. *et al.* Red cell adenylate kinase deficiency: molecular study of 3 new mutations (118G>A, 190G>A, and GAC deletion) associated with hereditary nonspherocytic hemolytic anemia. Blood 102, 353–356 (2003). 12649162 10.1182/blood-2002-07-2288

[b3] Dzeja P. P. & Terzic A. Adenylate Kinase and AMP Signaling Networks: Metabolic Monitoring, Signal Communication and Body Energy Sensing. Int. J. Mol. Sci. 10, 1729–1772 (2009). 19468337 10.3390/ijms10041729 PMC2680645

[b4] Hardie D. G. & Hawley S. A. AMP-activated protein kinase: the energy charge hypothesis revisited. BioEssays 23, 1112–1119 (2001). 11746230 10.1002/bies.10009

[b5] Wolf-Watz M. *et al.* Linkage between dynamics and catalysis in a thermophilic-mesophilic enzyme pair. Nat. Struc. Mol. Biol. 11, 945–949 (2004). 10.1038/nsmb821 15334070

[b6] Shapiro Y. E., Kahana E. & Meirovitch E. Domain Mobility in Proteins from NMR/SRLS. J. Phys. Chem. B 113, 12050–12060 (2009). 19673471 10.1021/jp901522c

[b7] Arora K. & Brooks C. L. III Large-scale allosteric conformational transitions of adenylate kinase appear to involve a population-shift mechanism. Proc. Natl. Acad. Sci. USA 104, 18496–18501 (2007). 18000050 10.1073/pnas.0706443104 PMC2141805

[b8] Maragakis P. & Karplus M. Large Amplitude Conformational Change in Proteins Explored with a Plastic Network Model: Adenylate Kinase. J. Mol. Biol. 352, 807–822 (2005). 16139299 10.1016/j.jmb.2005.07.031

[b9] Schulz G. E. Induced-fit movements in adenylate kinases. Faraday Discuss. 93, 85–93 (1992). 1290942 10.1039/fd9929300085

[b10] Bilderback T., Fulmer T., Mantulin W. W. & Glaser M. Substrate Binding Causes Movement in the ATP Binding Domain of Escherichia coli Adenylate Kinase. Biochemistry 35, 6100–6106 (1996). 8634252 10.1021/bi951833i

[b11] Müller C. W. & Schulz G. E. Structure of the complex between adenylate kinase from Escherichia coli and the inhibitor Ap5A refined at 1.9 Å resolution: A model for a catalytic transition state. J. Mol. Biol. 224, 159–177 (1992). 1548697 10.1016/0022-2836(92)90582-5

[b12] Jana B., Adkar B. V., Biswas R. & Bagchi B. Dynamic coupling between the LID and NMP domain motions in the catalytic conversion of ATP and AMP to ADP by adenylate kinase. J. Chem. Phys. 134, 035101 (2011). 21261390 10.1063/1.3516588

[b13] Daily M. D., Phillips Jr G. N. & Cui Q. Many Local Motions Cooperate to Produce the Adenylate Kinase Conformational Transition. J. Mol. Biol. 400, 618–631 (2010). 20471396 10.1016/j.jmb.2010.05.015 PMC2902635

[b14] Olsson U. & Wolf-Watz M. Overlap between folding and functional energy landscapes for adenylate kinase conformational change. Nat. Commun. 1, 111 (2010). 21081909 10.1038/ncomms1106

[b15] Whitford P. C., Miyashita O., Levy Y. & Onuchic J. N. Conformational Transitions of Adenylate Kinase: Switching by Cracking. J. Mol. Biol. 366, 1661–1671 (2007). 17217965 10.1016/j.jmb.2006.11.085 PMC2561047

[b16] Lou H. & Cukier R. I. Molecular Dynamics of Apo-Adenylate Kinase: A Distance Replica Exchange Method for the Free Energy of Conformational Fluctuations. J. Phys. Chem. B 110, 24121–24137 (2006). 17125384 10.1021/jp064303c

[b17] Maragakis P., Spichty M. & Karplus M. Optimal Estimates of Free Energies from Multistate Nonequilibrium Work Data. Phys. Rev. Lett. 96, 100602 (2006). 16605720 10.1103/PhysRevLett.96.100602

[b18] Miyashita O., Onuchic J. N. & Wolynes P. G. Nonlinear elasticity, proteinquakes, and the energy landscapes of functional transitions in proteins. Proc. Natl. Acad. Sci. USA 100, 12570–12575 (2003). 14566052 10.1073/pnas.2135471100 PMC240658

[b19] Ådén J. & Wolf-Watz M. NMR Identification of Transient Complexes Critical to Adenylate Kinase Catalysis. J. Am. Chem. Soc. 129, 14003–14012 (2007). 17935333 10.1021/ja075055g

[b20] Sheng X. R., Li X. & Pan X. M. An Iso-random Bi Bi Mechanism for Adenylate Kinase. J. Biol. Chem. 274, 22238–22242 (1999). 10428790 10.1074/jbc.274.32.22238

[b21] Müller C. W., Schlauderer G. J., Reinstein J. & Schulz G. E. Adenylate kinase motions during catalysis: an energetic counterweight balancing substrate binding. Structure 4, 147–156 (1996). 8805521 10.1016/s0969-2126(96)00018-4

[b22] Hanson J. A. *et al.* Illuminating the mechanistic roles of enzyme conformational dynamics. Proc. Natl. Acad. Sci. USA 104, 18055–18060 (2007). 17989222 10.1073/pnas.0708600104 PMC2084295

[b23] Henzler-Wildman K. A. & Kern D. Dynamic personalities of proteins. Nature 450, 964–972 (2007). 18075575 10.1038/nature06522

[b24] Potoyan D. A., Zhuravlev P. I. & Papoian G. A. Computing Free Energy of a Large-Scale Allosteric Transition in Adenylate Kinase Using All Atom Explicit Solvent Simulations. J. Phys. Chem. B 116, 1709–1715 (2012). 22212071 10.1021/jp209980b

[b25] Matsunaga Y. *et al.* Minimum Free Energy Path of Ligand-Induced Transition in Adenylate Kinase. PLos Comput. Biol. 8, e1002555 (2012). 22685395 10.1371/journal.pcbi.1002555 PMC3369945

[b26] Kubitzki M. B. & de Groot B. L. The Atomistic Mechanism of Conformational Transition in Adenylate Kinase: A TEE-REX Molecular Dynamics Study. Structure 16, 1175–1182 (2008). 18682219 10.1016/j.str.2008.04.013

[b27] Krishnamurthy H., Lou H., Kimple A., Vieille C. & Cukier R. I. Associative mechanism for phosphoryl transfer: A molecular dynamics simulation of Escherichia coli adenylate kinase complexed with its substrates. Proteins: Struc. Funct. Bioinf. 58, 88–100 (2005). 10.1002/prot.20301 15521058

[b28] Wang Y., Gan L., Wang E. & Wang J. Exploring the Dynamic Functional Landscape of Adenylate Kinase Modulated by Substrates. J. Chem. Theory Comput. 9, 84–95 (2013). 10.1021/ct300720s 26589012

[b29] Pontiggia F., Zen A. & Micheletti C. Small- and Large-Scale Conformational Changes of Adenylate Kinase: A Molecular Dynamics Study of the Subdomain Motion and Mechanics. Biophys. J. 95, 5901–5912 (2008). 18931260 10.1529/biophysj.108.135467 PMC2599863

[b30] Henzler-Wildman K. A. *et al.* A hierarchy of timescales in protein dynamics is linked to enzyme catalysis. Nature 450, 838–844 (2007). 18026087 10.1038/nature06407

[b31] Beckstein O., Denning E. J., Perilla J. R. & Woolf T. B. Zipping and Unzipping of Adenylate Kinase: Atomistic Insights into the Ensemble of Open ↔ Closed Transition. J. Mol. Biol. 394, 160–176 (2009). 19751742 10.1016/j.jmb.2009.09.009 PMC2803350

[b32] Bonomi M. *et al.* PLUMED: A portable plugin for free-energy calculations with molecular dynamics. Comput. Phys. Commun. 180, 1961–1972 (2009).

[b33] Bonomi M., Barducci A. & Parrinello M. Reconstructing the equilibrium Boltzmann distribution from well-tempered metadynamics. J. Comput. Chem. 30, 1615–1621 (2009). 19421997 10.1002/jcc.21305

[b34] Barducci A., Bussi G. & Parrinello M. Well-Tempered Metadynamics: A Smoothly Converging and Tunable Free-Energy Method. Phys. Rev. Lett. 100, 020603 (2008). 18232845 10.1103/PhysRevLett.100.020603

[b35] Branduardi D., Gervasio F. L. & Parrinello M. From A to B in free energy space. J. Chem. Phys. 126, 054103 (2007). 17302470 10.1063/1.2432340

[b36] Grazioso G. *et al.* Investigating the Mechanism of Substrate Uptake and Release in the Glutamate Transporter Homologue GltPh through Metadynamics Simulations. J. Am. Chem. Soc. 134, 453–463 (2012). 22092197 10.1021/ja208485w

[b37] Limongelli V. *et al.* Sampling protein motion and solvent effect during ligand binding. Proc. Natl. Acad. Sci. USA 109, 1467–1472 (2012). 22238423 10.1073/pnas.1112181108 PMC3277130

[b38] Song H. D. & Zhu F. Conformational Dynamics of a Ligand-Free Adenylate Kinase. PLos ONE 8, e68023 (2013). 23861846 10.1371/journal.pone.0068023 PMC3702565

[b39] Dahnke T., Shi Z., Yan H., Jiang R. & Tsai M. Mechanism of adenylate kinase. Structural and functional roles of the conserved arginine-97 and arginine-132. Biochemistry 31, 6318–6328 (1992). 1627570 10.1021/bi00142a022

[b40] Reinstein J. *et al.* Structural and catalytic role of arginine 88 in Escherichia coli adenylate kinase as evidenced by chemical modification and site-directed mutagenesis. J. Biol. Chem. 264, 8107–7112 (1989). 2542263

[b41] Byeon I. L., Shi Z. & Tsai M. Mechanism of Adenylate Kinase. The "Essential Lysine" Helps To Orient the Phosphates and the Active Site Residues to Proper Conformations. Biochemistry 34, 3172–3182 (1995). 7880812 10.1021/bi00010a006

[b42] Reinstein J., Brune M. & Wittinghofer A. Mutations in the nucleotide binding loop of adenylate kinase of Escherichia coli. Biochemistry 27, 4712–4720 (1988). 2844237 10.1021/bi00413a020

[b43] Hornak V. *et al.* Comparison of Multiple Amber Force Fields and Development of Improved Protein Backbone Parameters. Proteins: Struc. Funct. Bioinf. 65, 712–725 (2006). 10.1002/prot.21123 PMC4805110 16981200

[b44] Cornell W. D. *et al.* A Second Generation Force Field for the Simulation of Proteins, Nucleic Acids, and Organic Molecules. J. Am. Chem. Soc. 117, 5179–5197 (1995).

[b45] Jorgensen W. L., Chandrasekhar J., Madura J. D., Impey R. W. & Klein M. L. Comparison of simple potential functions for simulating liquid water. J. Chem. Phys. 79, 926–935 (1983).

[b46] Hess B., Kutzner C., van der Spoel D. & Lindahl E. GROMACS 4: Algorithms for Highly Efficient, Load-Balanced, and Scalable Molecular Simulation. J. Chem. Theory Comput. 4, 435–447 (2008). 10.1021/ct700301q 26620784

[b47] Essmann U. *et al.* A smooth particle mesh Ewald method. J. Chem. Phys. 103, 8577–8593 (1995).

[b48] Darden T., York D. M. & Pedersen L. Particle mesh Ewald: An N·log(N) method for Ewald sums in large systems. J. Chem. Phys. 98, 10089–10092 (1993).

[b49] Hess B. P-LINCS: A Parallel Linear Constraint Solver for Molecular Simulation. J. Chem. Theory Comput. 4, 116–122 (2008). 10.1021/ct700200b 26619985

[b50] Bussi G., Donadio D. & Parrinello M. Canonical sampling through velocity rescaling. J. Chem. Phys. 126, 014101 (2007). 17212484 10.1063/1.2408420

[b51] Laio A. & Parrinello M. Escaping free-energy minima. Proc. Natl. Acad. Sci. USA 99, 12562–12566 (2002). 12271136 10.1073/pnas.202427399 PMC130499

[b52] Di Leva F., Novellino E., Cavalli A., Parrinello M. & Limongelli V. Mechanistic insight into ligand binding to G-quadruplex DNA. Nucl. Acids Res. 42, 5447–5455 (2014). 24753420 10.1093/nar/gku247 PMC4027208

[b53] Limongelli V., Bonomi M. & Parrinello M. Funnel metadynamics as accurate binding free-energy method. Proc. Natl. Acad. Sci. U. S. A. 110, 6358–6363 (2013). 23553839 10.1073/pnas.1303186110 PMC3631651

[b54] Ren W., Vanden-Eijnden E., Maragakis P. & Weinan E. Transition pathways in complex systems: Application of the finite-temperature string method to the alanine dipeptide. J. Chem. Phys. 123, 134109 (2005). 16223277 10.1063/1.2013256

[b55] Humphrey W., Dalke A. & Schulten K. VMD: Visual molecular dynamics. J. Mol. Graph. 14, 33–38 (1996). 8744570 10.1016/0263-7855(96)00018-5

