# Supplementary material for: Energetics and Structural Characterization of the large-scale Functional Motion of Adenylate Kinase
Source: Sci Rep. 2015 Feb 12;5:8425. doi: 10.1038/srep08425 (PMC4325324; doi:10.1038/srep08425)
Supplement: Supplementary Information [file srep08425-s1.pdf]

SUPPORTING INFORMATION

Energetics and Structural Characterization of the large-scale Functional Motion of Adenylate Kinase

Elena Formoso[1,2], Vittorio Limongelli[3,4] and Michele Parrinello[1]

[1]*Department of Chemistry and Applied Biosciences, ETH Zurich , and Faculty of Informatics, Institute of Computational Science, Università della Svizzera Italiana, via G. Buffi 13, CH-6900 Lugano, Switzerland*

[2]*Kimika Fakultatea, Euskal Herriko Unibertsitatea (UPV/EHU) and Donostia International Physics Center (DIPC), PK 1072, 20080 Donostia, Euskadi, Spain*

[3] *Università della Svizzera Italiana (USI), Faculty of Informatics, Institute of Computational Science, via G. Buffi 13, CH-6900 Lugano, Switzerland*

[4]*Department of Pharmacy, University of Naples "Federico II", via D. Montesano 49, I-80131 Naples, Italy*

To whom correspondence may be addressed: vittoriolimongelli@gmail.com or parrinello@phys.chem.ethz.ch

Supporting Information List:

- The results of the standard MD simulations carried out on the closed and open states of the ligand-free Adenylate Kinase. (Figures S1 and S2)
- The residues used to define the path collective variables (Table S1)
- rmsd and rmsf values of the well-tempered metadynamics simulation (Figures S3 and S4)
- The FES associated with the rmsd respected to the X-ray structures (Figure S5)
- The differences in the inter-residue interactions between X-ray and well-tempered metadynamics structures (Figure S6)
- Supplementary video legend

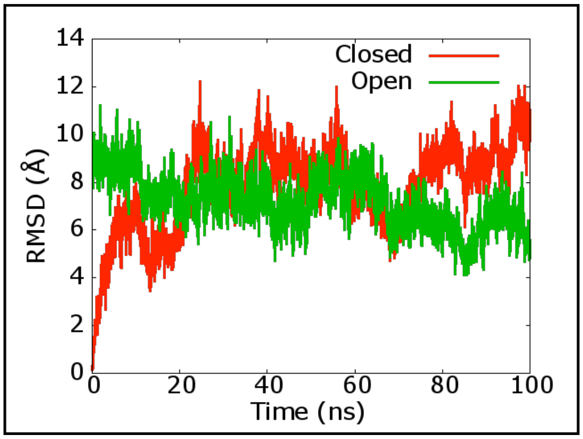

Figure S1: rmsd values of the closed (red) and open (green) ligand-free AK systems' backbone atoms vs the fully-ligated closed X-ray structure.

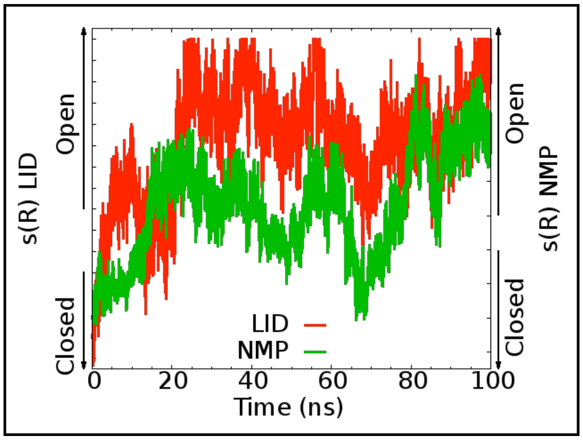

Figure S2: LID (red) and NMP (green) domain motion of the ligand-free AK in the closed state during MD simulation represented as a function of simulation time. The motion of the domains is represented by the s(R) path CV.

Table S1: List of residues which forms the LID and NMP domains path.

		Residues
LID	Aligned	105, 107, 109, 111, 113, 114, 176-178, 180, 182, 184, 186, 188
	rmsd	113, 114, 118, 123-126, 131-134, 153, 155, 156, 162, 165, 176, 177
NMP	Aligned	1-36, 61, 63, 65, 67-117, 161-214
	rmsd	6, 20, 24, 26-37, 40, 44, 47, 50, 52, 55, 61, 63, 65, 67-69, 71, 81, 84, 91, 95, 96

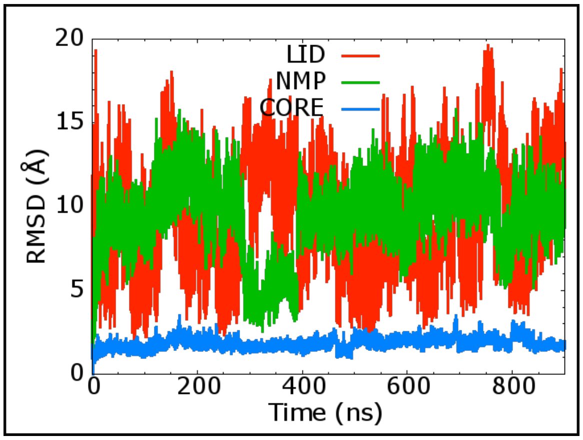

Figure S3: rmsd values calculated for each domain of AK undergone to well-tempered metadynamics simulations. All the structures were aligned using the CORE domain's Cα atoms. The LID is depicted in red, NMP is depicted in green and CORE is shown in blue.

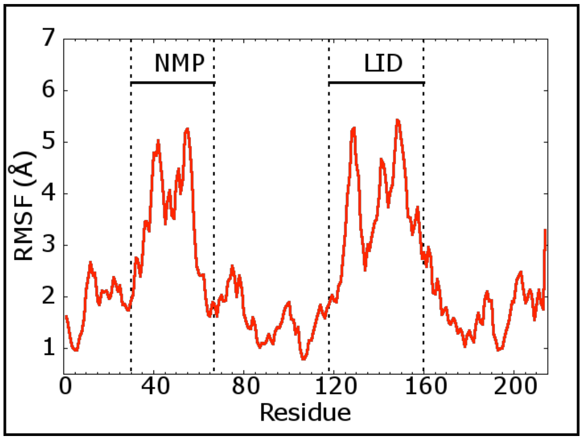

Figure S4: rmsf values calculated for all the AK residues undergone to well-tempered metadynamics simulations.

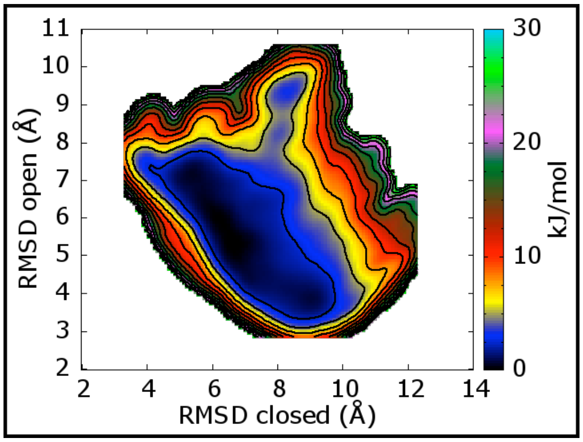

Figure S5: The FES of AK motion reconstructed as a function of the rmsd values calculated with respect to the X-ray structures of the closed ligand-bound enzyme[1] and open ligand-free enzyme.[2] The separation between contours is 2.5 $kJmol^{-1}$.

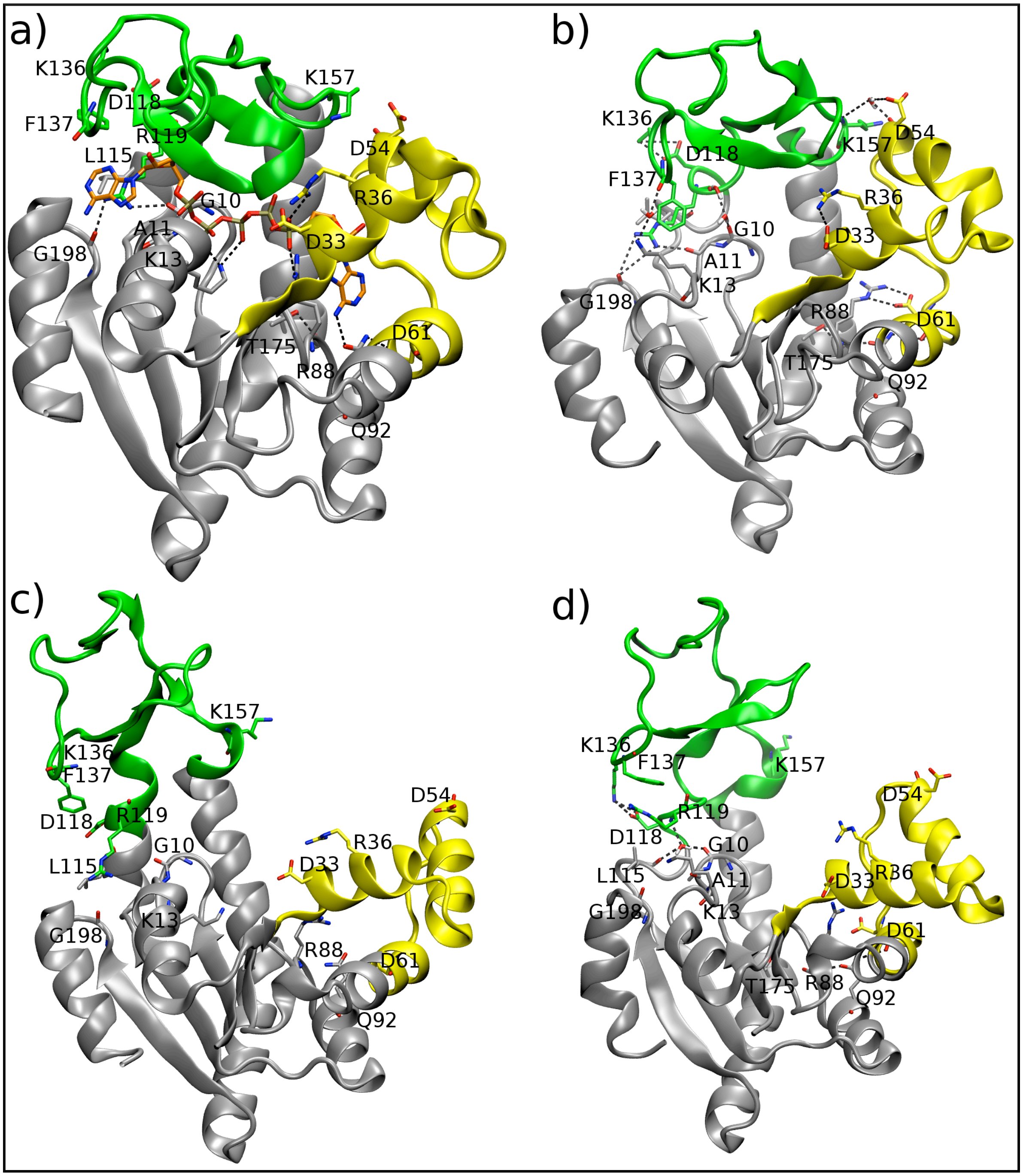

Figure S6: Inter-residue interactions for: a) closed X-ray structure, 1AKE;[1] b) closed metadynamics state (minimum A); c) open X-ray structure, 4AKE;[2] and d) open metadynamics state (minimum B). We note that the open X-ray structure was obtained as crystallographic dimer where the position of the LID and NMP domains in one monomer influences their location

in the other. The LID and NMP domains are colored in green and yellow, respectively. The co-crystallized inhibitor, mimicking the two physiological substrates, is colored in orange.

The figures were rendered using the VMD software[3] while the graphs were generated using gnuplot.

Supplementary video legend. The movie of the opening/closing functional motion of AK under the action of metadynamics.

References

[1] Müller, C.W. & Schulz, G.E. Structure of the complex between adenylate kinase from Escherichia coli and the inhibitor Ap5A refined at 1.9 Å resolution: A model for a catalytic transition state. *J. Mol. Biol.* **224**, 159-177 (1992).

[2] Müller, C.W., Schlauderer, G.J., Reinstein, J. & Schulz, G.E. Adenylate kinase motions during catalysis: an energetic counterweight balancing substrate binding. *Structure* **4**, 147-156 (1996).

[3] W. Humphrey, A. Dalke, K. Schulten, *J. Mol. Graph.* **1996**, *14*, 33-38.
